# Are Dolphins Kept in Impoverished Environments?

**DOI:** 10.3390/ani13172707

**Published:** 2023-08-25

**Authors:** Kelly Jaakkola

**Affiliations:** Dolphin Research Center, Grassy Key, FL 33050, USA; kelly@dolphins.org

**Keywords:** dolphins, cetacea, captivity, animal welfare, impoverishment, cognitive enrichment

## Abstract

**Simple Summary:**

Dolphins are among the most popular animals in zoos and aquariums around the world. Recently, however, a paper suggested that environments for dolphins in zoos and aquariums might be “impoverished”, leading to possible problems for their brain and cognitive functioning. This review directly examines that hypothesis in light of the existing scientific literature relevant to dolphin welfare in zoos and aquariums. The results of this analysis—based on the documented standards of modern professional zoological organizations, the results of a multi-national study on dolphin welfare, and the behavior of dolphins in modern zoos and aquariums—show that this claim is clearly false. However, as “not impoverished” would be a ridiculously low bar to set as an animal welfare standard, additional strategies are suggested for providing cognitive challenges in zoos and aquariums to optimize dolphins’ cognitive well-being.

**Abstract:**

Numerous studies have demonstrated the negative effects of impoverished environments versus the positive effects of enriched environments on animals’ cognitive and neural functioning. Recently, a hypothesis was raised suggesting that conditions for dolphins in zoological facilities may be inherently impoverished, and thus lead to neural and cognitive deficits. This review directly examines that hypothesis in light of the existing scientific literature relevant to dolphin welfare in zoological facilities. Specifically, it examines how dolphins are housed in modern zoological facilities, where the characteristics of such housing fall on the continuum of impoverished-to-enriched environments, and the extent to which dolphins show behavioral evidence characteristic of living in impoverished environments. The results of this analysis show that contrary to the original hypothesis, modern zoological facilities do not inherently, or even typically, house dolphins in impoverished conditions. However, it also notes that there is variation in animal welfare across different zoological facilities, and that “not impoverished” would be a particularly low bar to set as an animal welfare standard. To optimize cognitive well-being, strategies for providing additional cognitive challenges for dolphins in zoological facilities are suggested.

## 1. Introduction

Around the world, bottlenose dolphins are among the most popular species, belonging to the group of animals known as “charismatic megafauna”, to which people feel particularly strong emotional connections [1,2,3,4,5]. At least part of this popularity is likely due to the public’s experience with this species in zoological facilities (i.e., zoos, aquaria, and marine mammal facilities), where they are the most common species of cetacean displayed [6,7], and where their high rates of activity and interaction with humans make them particularly appealing [8,9,10,11].

Research over the past couple of decades suggests that dolphins typically fare well in modern zoological facilities, with life spans as long as or longer than in the wild [12], stress levels as reflected in baseline cortisol no higher than in the wild [13,14], high reproductive rates [15,16], high adaptability [7], and generally good welfare [17]. Recently, however, a paper by Jacobs et al. [18] challenged this view with a controversial hypothesis that living in modern zoos and aquaria might cause neural deficits in dolphins (as well as in other cetaceans and elephants). Specifically, they argue that because it has been shown that housing animals in impoverished environments results in a number of negative neural consequences, housing dolphins in zoological facilities should result in these same negative neural consequences.

The detrimental effects of impoverished environments on neural and cognitive functioning are noncontroversial, as numerous studies have demonstrated the cognitive and neural effects of housing animals in enriched (positive effects) versus impoverished (negative effects) environments (see reviews in [19,20,21,22,23]). It is puzzling, however, why Jacobs et al. [18] would choose to recast this issue as reflecting an inherent difference between animals in the wild versus in zoological facilities, when that entire field of scientific literature examines the effects of different levels of enrichment within zoological facilities and other human-controlled environments (e.g., homes, laboratories) (see references in Jacobs et al. [18]). In reframing this issue to consistently conflate “captive” with “impoverished” environments, Jacobs et al. ignore the last 45 years of scientific literature on animal welfare and the effects of various enrichment practices in zoological settings [24,25,26,27,28]. Be that as it may, the serious allegation they raise calls for serious discussion. The validity of their hypothesis (i.e., that impoverished environments cause neural deficits in other animals, therefore zoos cause neural deficits in dolphins) directly depends on the extent to which the zoological facilities where dolphins are housed embody impoverished environments.

In the current paper, we directly examine this proposed equivalence in light of the existing scientific literature relevant to dolphin welfare in zoological facilities. Specifically, we review the following: (a) how dolphins are housed in modern zoological facilities, (b) where the characteristics of such housing fall on the continuum of impoverished-to-enriched environments described by Jacobs et al. [18], and (c) the extent to which dolphins show behavioral evidence characteristic of living in impoverished environments.

## 2. What Do We Mean by “Impoverished”?

In the standard experimental framework used in hundreds of experiments to explore the neural and cognitive effects of different environments, animals are housed in one of three conditions: impoverished, standard/control, or enriched/complex. Then, after a period of time, the animals from two or more of these contrasting conditions are compared on a cognitive task, or their brains are compared on a neural characteristic. As summarized by Jacobs et al. [18], these three types of conditions can typically be characterized as follows:

- Impoverished = animals are alone in smaller cages with no opportunity for social or object interaction;
- Standard/control = several animals are housed together without opportunities to interact with stimulatory objects;
- Enriched/complex = animals are housed in a relatively large enclosure together with several conspecifics and multiple objects/toys, which are frequently changed to provide novelty.

As described above, the types of environments in these studies vary along three factors: (a) social situation (whether the animal is solitary or with others); (b) size of enclosure; and (c) complexity and variability of the environment. The levels of these factors are not independently varied, but rather each varies along a similar but not identical continuum across the environments, as shown in Table 1.

With this framework in mind, we are now in a position to examine the conditions in which dolphins are housed in modern zoological facilities, with particular attention to these three factors.

## 3. Dolphin Environments in Modern Zoological Facilities

Jacobs et al. [18] argue that zoological facilities are inherently impoverished environments for dolphins, framing their argument as the effects of “captivity” as a whole, and repeatedly using the conflated term “captive/impoverished” (or “impoverished/captive”). Notably, their claim is not simply that some dolphins are housed in impoverished conditions, but rather that the conditions in zoological facilities are *inherently* impoverished. This is an important distinction. If poor conditions at some zoological facilities are negatively impacting animal welfare, then the solution to poor welfare is to improve the conditions in those facilities. However, if variation between zoological facilities is irrelevant because conditions at even the best facilities are inherently impoverished, then the only possible solution to poor welfare is to not have dolphins in zoological facilities at all.

Professional zoological organizations—including the Alliance of Marine Mammal Parks and Aquariums (AMMPA), the Association of Zoos and Aquariums (AZA), the European Association of Zoos and Aquaria (EAZA), and the European Association for Aquatic Mammals (EAAM)—provide standards and guidelines that govern the housing conditions and management practices in every zoological facility accredited by that organization [29,30,31,32]. In the United States and Europe, approximately 75% of facilities housing dolphins belong to at least one of these organizations [33,34,35,36,37]. Thus, we can use the standards and guidelines of these organizations to examine the relevant practices typical of facilities in these jurisdictions.

### 3.1. Social Situation

According to Jacobs et al.’s [18] framework, in an impoverished environment, the animals are housed alone. In contrast, the standards of professional zoological organizations stipulate that dolphins must be housed in appropriate social groupings, only isolated when strictly necessary for the animal’s health or well-being [30,31,32].

### 3.2. Enclosure Size

According to Jacobs et al.’s [18] framework, in an impoverished environment, the animals are housed in small enclosures. There is no agreed-upon objective measurement defining what qualifies as “small”, however, other than being smaller than the other enclosures to which it is being compared. Similarly, the standards of professional zoological organizations require that dolphin enclosures provide sufficient space to meet the animals’ physical and social needs [29,30,31,32]; however, there is no universally agreed-upon standard as to how large that space should be. Deciding on this issue scientifically can be difficult, as numerous animal welfare experts have noted that it is the quality, rather than quantity, of the space that may be most important for animal well-being [38,39,40]. Ideally, then, enclosure size requirements for any particular species should be informed by studies of how the size, quality, and management of the space impact welfare outcomes for that species. Recently, such a series of studies was conducted as part of a large, multi-institutional collaborative research effort (called the Cetacean Welfare Study) undertaken among zoological facilities accredited by AMMPA and AZA (see [41] and associated papers).

Two of the biggest concerns with small enclosures are that they may: (a) restrict the animals’ ability to perform normal behaviors and achieve typical and healthy levels of activity and exercise, and (b) increase aggression due to forced proximity while simultaneously restricting animals’ ability to retreat from aggressive situations [18,40,42,43].

#### 3.2.1. Activity Levels

The only study to date that has directly compared activity levels of the same dolphins in different-sized pools seems to give credence to this concern. Bassos and Wells [44] reported that two dolphins swam significantly more when housed in a larger pool (a 54 × 40 ft oval), and were inactive significantly more when housed in a smaller pool (a 30 ft diameter circle). It is worth noting, however, that both pools in that study were significantly smaller (i.e., the longest horizontal distance for the larger pool was 2.5 times smaller) than the average size of enclosures in the Cetacean Welfare Study [45]. Thus, while there is evidence that particularly small enclosures can negatively impact activity levels, this enclosure size is not representative of dolphin enclosures in modern zoological facilities.

In addition, several studies have reported that dolphins kept in larger sea pen enclosures swim more than dolphins that are kept in smaller pool enclosures [45,46,47,48]. However, since these enclosures differ in both size and complexity/variability, it is unclear which variable is responsible for this difference in activity (see Section 3.3 on environmental complexity and variability). Representative data on activity levels in modern zoological facilities can be seen in the Cetacean Welfare Study, which found that dolphins swam an average of 2.32 km each hour outside of training sessions [49]. For comparison, studies of wild dolphins have reported travel distances of up to 30 to 50.2 km in a day [50,51,52], which translates to an average of 1.25 to 2.09 km each hour. These numbers may not be directly analogous, given differences in sampling times and methodologies between the two types of environments; however, the data do not show any evidence of a negative impact on activity levels for dolphins in modern zoological facilities.

#### 3.2.2. Aggression

The only study to date that has directly examined the aggressive behavior of dolphins in different-sized pools also gives credence to the concern about particularly small enclosures. Specifically, Myers and Overstrom [53] reported that dolphins displayed a large number of sexual/aggressive responses when they were kept in a small holding pool between shows. It is worth noting, however, that the 7.3 m diameter holding pool was actually divided by nets into four quadrants in which each of the four dolphins was kept individually [53,54]. In this arrangement, the longest horizontal distance for each quadrant was approximately eight times smaller than the average for the enclosures in the Cetacean Welfare Study [45]. Therefore, while there is evidence that particularly small enclosures with physically isolated animals can increase aggression, this enclosure size and social situation are not at all representative of dolphin enclosures in modern zoological facilities. In fact, once the net barriers were removed and the animals were housed together in the holding pool, the sexual/aggressive response ceased occurring [53]. A more representative picture of aggression in modern facilities can be seen in the Cetacean Welfare Study, which found levels of overt aggression among bottlenose dolphins to be quite low [55].

### 3.3. Complexity and Variability of the Environment

According to Jacobs et al.’s [18] framework, in an impoverished environment the animals are housed in barren enclosures with no objects to interact with and no variability. In contrast, the standards of professional zoological organizations stipulate that dolphins’ housing must provide environmental complexity and enrichment [29,30,31,32]. With respect to the current discussion, it is worth noting that any environmental complexity or enrichment at all would be enough to elevate the level of this factor above “impoverished”. However, with respect to the broader issues regarding animal welfare, it is worth pointing out that the practice of environmental enrichment is much more elaborate than this criterion suggests [24,27,28].

In practice, zoological facilities differ with respect to the type(s) of enrichment provided to dolphins, how much variability there is in the enrichment schedule, and how frequently new types of enrichment are introduced [45]. These differences are then reflected in multiple indicators of animal welfare, including habitat use, activity levels, and positive social interactions [41,49,55,56]. In fact, consistent with previous research on terrestrial animals [57,58,59,60], the Cetacean Welfare Study found that enrichment programs were more important to dolphin welfare than was the size of their enclosure [41].

In addition to environmental features and objects, the positive reinforcement training sessions that are standard for dolphins in zoological facilities likely provide additional enrichment [43,61,62,63,64,65,66]. Different from older traditional training techniques that may rely on coercion and punishment, positive reinforcement training focuses on the timing of providing various reinforcers (e.g., food, praise, rubs) dependent on the animal’s behavior in order to encourage specific behaviors over others [67,68]. By gradually changing the criteria for which behaviors get reinforced, the trainer can lead the animal through a series of successive approximations to a target behavior. To be clear, this technique does not require withholding food [63,69]. Rather, the trainer makes precise moment-to-moment decisions regarding exactly when*—*not whether*—*to provide reinforcers. Performed correctly, this kind of training provides animals with mental stimulation [43,63,67], increases their physical activity and behavioral variability [62,63,67,70], and increases opportunities for choice and control over aspects of their environment [43,63,64,66].

### 3.4. Impoverished, Standard, or Enriched?

The results of the above analysis suggest that, contrary to Jacobs et al.’s [18] hypothesis, modern zoological facilities do not inherently, or even typically, house dolphins in impoverished conditions. If they did, then according to Jacobs et al.’s definitions, the dolphins would be housed alone, in small enclosures, with no objects or other enriching experiences (see Table 1). Instead, both the accreditation standards of professional zoological organizations and the results of a large multi-national study on cetacean welfare showed that dolphins in zoological facilities today are normally housed in social groups, with a variety of enriching objects and experiences, in enclosures much larger than the abnormally small pools referenced as problematic in earlier studies. Rather than impoverished, a more accurate description of these environments would thus place them at the level of standard/control or enriched/complex (Table 1).

## 4. Do Dolphins Show Any Behavioral Evidence of Living in Impoverished Environments?

In spite of the preceding analysis, one might theoretically argue that even if the conditions of their housing in modern zoological facilities do not fit the classic definition of impoverished environments, it is possible that dolphins could have particularly demanding cognitive and social needs, and that therefore what counts as “impoverished” (i.e., leading to detrimental effects) might fall at a different point on the continuum. To explore this possibility, we next examine whether dolphins in modern zoological facilities routinely exhibit behavioral characteristics suggesting the detrimental effects of living in impoverished environments. Specifically, Jacobs et al. [18] propose that cetaceans in facilities exhibit three general behavioral/psychological characteristics—stereotypies, hyperaggression, and symptoms characteristic of depression—that appear to be “manifestations of the same kinds of neurobiological deficits demonstrated in other mammals in impoverished environments” (p. 443). As before, the relevant question is not whether any dolphin in any facility has ever exhibited these characteristics (which would be an individual welfare problem that needs to be addressed), but rather whether this is the normal state for dolphins in modern facilities, as would be expected if zoological facilities are inherently an impoverished situation for dolphins.

### 4.1. Stereotypies

Stereotypies are generally defined as invariant, repetitive behavior patterns that have no obvious goal or function [71,72]. These behaviors can take many forms, such as rocking or weaving [73,74], pacing [75,76], or repetitive jumping [77,78]. Note that it is typically not the specific behavioral unit that is considered problematic—nobody would be the least concerned about an animal that shifted their weight, walked across the front of their enclosure, or jumped. Rather, it is the largely invariant and perseverative repetition that marks the behavior as aberrant.

The existence of stereotypical behavior has long been thought to indicate that an animal’s environment is deficient or inadequate in some way (e.g., [71,79]), as suggested by a multitude of studies showing that housing animals in impoverished environments is associated with the development of stereotypies (reviewed in, e.g., [71,80]), while enriching an animal’s environment often reduces stereotypies (reviewed in, e.g., [81,82]). One complicating factor with this interpretation is that stereotypies can persist even after the animal is moved from an impoverished to an enriched environment, not unlike a psychological scar indicating the effects of a past situation [80,83]. For current purposes, then, it seems fair to conclude that if stereotypical behaviors are prevalent, this would likely indicate some deficiency in the animals’ current or past environment.

To be sure, stereotypical behavior has been—and continues to be—an ongoing issue with many animals in various types of human-controlled environments, occurring, for example, in an estimated 18% of horses housed in stables, 82% of wild carnivores in zoos, and 91% of pigs (sows) kept in gestation crates [80]. This suggests that even with the considerable advancements in animal welfare science and practice over the past several decades in zoos, farms, and laboratories [84,85,86,87,88], there is still a need for even more widespread attention to environmental complexity and enrichment for animal species under all types of human care (see Section 5 on optimizing cognitive well-being below).

With respect to dolphins, several decades ago, Greenwood [89] reported a clear case of stereotyped “head-pressing” behavior in three individual bottlenose dolphins, in which they would repeatedly press their foreheads against the wall of their pools. The enclosures in which the dolphins were kept during this time were extremely small and barren. Specifically, the largest was 60,000 L, which is smaller than the minimum size required by professional zoological standards [29,30,31], and would be illegal in many countries today [90]. When the dolphins were moved to larger pools, this stereotypy immediately disappeared [89]. This tells us that dolphins, like many other animals, exhibit stereotypies in problematic environments. So are stereotypies a prevalent issue with dolphins in modern zoological facilities?

For bottlenose dolphins, the behavior most often suggested as a potential stereotypy is repetitively swimming in a fixed pattern around their pool [42,47,91,92,93]. To that end, studies have found that dolphins in facilities do spend a considerable amount of time swimming around the perimeter of their enclosures [47,92,94], perhaps especially when the enclosures are smaller [47,92]. However, a number of researchers have questioned whether this is actually a stereotypy (e.g., [42,91,95]). Importantly, a hallmark of stereotypic route tracing (such as stereotypic pacing or swimming) is a dramatic invariance in the animal’s motor pattern, such as turning at the same location, placing their feet at the same spot, and/or taking the same number of steps in their route [76,96,97]. When viewed through this lens, the one study that coded aspects of the dolphins’ behavior in addition to their route [92] found that although the dolphins swam in clockwise circles around their pool, the “routes of the swimming patterns were not followed very strictly” (p. 342), and that “turning, breathing and leaping were not shown consistently at any particular locations” (p. 340). Similarly, more recent studies that incorporated this notion of a relatively invariant path found that stereotypic route tracing happened extremely infrequently, occurring in less than 1% of scans in a study of 18 dolphins in 6 facilities [98], and an average of 0.00 times per minute in a multi-national study of 47 dolphins at 25 facilities [99].

However, if perimeter swimming is not a stereotypical behavior, then why do dolphins do it? Several researchers have noted that it may actually serve a patrolling function [91,100]. However, this explanation overlooks the finding that perimeter swimming may be more common in smaller enclosures than in large ones [47,92]. A simpler explanation may stem from the fact that dolphins typically spend a large proportion of their time traveling [101,102,103]. In an enclosure, circling the perimeter is the closest approximation to sustained traveling without abrupt changes of direction for no reason. From this explanation, one would expect that dolphins who have the most linear space would swim linearly more, while dolphins with less linear space would circle the perimeter more (cf. [47]).

### 4.2. Hyperaggression

Despite Jacobs et al.’s [18] claim that “hyperaggression is more common in captive cetaceans than in their free counterparts” (pp. 442–443), there is in fact no scientific evidence to suggest that this is true. On the contrary, multiple studies have documented wild dolphins displaying aggressive and even lethal behavior towards adults and infants of their own and other cetacean species (see, e.g., [104,105,106,107,108,109,110,111,112,113]). This is not an aberration, but rather typical dominance, territorial, and reproduction-related behavior common in dolphins and other animal species [112,114,115,116]. It is precisely because social conflicts are both natural and expected that the standards of professional zoological organizations all require that facilities have strategies to monitor and mitigate potential conflict and incompatibility between animals, including things such as behavioral training, barriers, and possible separation when conflicts arise [29,30,31,32]. While there have been no studies as yet directly comparing aggression rates or severity of dolphins in the wild versus in zoological facilities, as noted earlier, a large, multi-institutional collaborative research study found that aggression among bottlenose dolphins in modern facilities is in fact quite low [55].

### 4.3. Symptoms Characteristic of Depression

In humans, depression is a complex, variable disorder characterized by feelings of sadness, a loss of interest or pleasure, changes in weight or appetite, feeling fatigued, a decreased ability to concentrate, sleep problems, psychomotor agitation or retardation, low self-confidence, feelings of worthlessness and guilt, and recurrent thoughts of death or suicide [117,118]. While depressed individuals may not show evidence of all of these characteristics, a diagnosis of depression requires the presence of at least five of these characteristics co-occurring for an extended period of time (i.e., at least two weeks), and excluding any characteristics that are attributable to other physical or medical causes [117,118].

Although some of these diagnostic criteria can only be assessed via verbal self-report (e.g., low self-confidence, feelings of worthlessness) and are thus only knowable for humans, other criteria can be, and have been, objectively assessed in other species, leading to well-established indicators of possible depression in non-human animals [117]. Within this context, Jacobs et al.’s [18] claim that some cetaceans “exhibit symptoms characteristic of depression (e.g., logging on the surface, lying motionless on the bottom of the tank, and loss of appetite)” (p. 442) is problematic on several levels. First, to use any characteristic (e.g., inactivity, loss of appetite) as an indicator of possible depression in an individual, one would need prevalence rates of that characteristic for that individual. How frequently the behavior occurs is the relevant concern. Second, one would need to be reasonably certain that the characteristic in question is not attributable to physical causes such as illness or old age. This is particularly important given that these particular characteristics (i.e., decrease in activity, loss of appetite) are specifically known to develop with illness and/or age in dolphins and other species [119,120,121,122]. Third, there would need to be a co-occurrence of several diagnostic criteria in the same individual over the same time period. The practice of relying on single behaviors is a known problem in animal research on depression-like states, which risks mislabeling animals as depressed when they are not [117]. Finally, to credibly argue that this is a population-level issue for animals in modern zoological facilities, one would need to show that this was prevalent in most, or at least many, of the animals in that environment. Jacobs et al. [18] present no data that address any of these criteria.

However, even if appetite loss and inactivity (including both floating/logging on the surface and resting on the bottom) do not necessarily indicate depression, it is worth noting that both of these measures have been used to assess individual health and welfare in a variety of animals (e.g., [123,124,125]). With respect to dolphins, for example, welfare studies have shown that how often dolphins receive new enrichment is associated with their activity rates (including how often they interact with other dolphins, how much they swim, and how much energy they expend) [49,55]; that interacting with a caregiver can lead to increases in activity (including playing with toys more and floating in place less) [126]; and that decreased appetite may be associated with stressful situations, disease, or other medical conditions (e.g., [119,120,121,127]). Such measures can thus be extremely useful for assessing the welfare of individual dolphins. However, there are no data suggesting that these are population-wide problems in modern zoological facilities as Jacobs et al. [18] suggest.

## 5. Optimizing Cognitive Well-Being

Thus far, we have been examining the existing evidence relevant to assessing Jacob et al.’s [18] claim that conditions for dolphins in zoological facilities are inherently impoverished (and thus lead to neural deficits). The results of this analysis show that this claim is clearly false. The accreditation standards of modern professional zoological organizations, the results of a large multi-national study on cetacean welfare, and the behavior of dolphins in modern zoological facilities all point to the same conclusion that modern zoological facilities do not inherently, or even typically, house dolphins in impoverished conditions.

That said, however, “not impoverished” would be a particularly low bar to set as an animal welfare standard. As noted in the introduction, numerous studies have documented not only the negative effects of impoverished environments, but also the positive effects of enriched environments on the neural and cognitive functioning of a variety of animals (see reviews in, e.g., [19,20,21,22,23]). Further, we have seen that even among accredited zoological facilities, there can be variation in welfare outcomes (e.g., [41,49,55,99]). To optimize the welfare of animals in their care, then, zoological facilities would be well-served to pay specific attention to environmental promoters of animals’ cognitive well-being [91,128,129]. To that end, beyond the three foundational factors already discussed (i.e., social situation, enclosure size, and environmental complexity/variability; see Table 1), we would like to briefly review additional practices that have been suggested to positively impact cognitive welfare.

### Beyond Simple Enrichment

Environmental enrichment can come in a variety of forms and complexity levels. For both aquatic and terrestrial animals, however, the most common type tends to be “toys” or other manipulable objects [42,45,91,130]. To be sure, many studies have shown positive benefits of this type of enrichment [81,82,131]. However, others have shown that such simple object enrichment may not be sufficient to produce long-lasting benefits due to animals’ lack of interest or habituation [132,133,134,135].

Beyond providing animals with objects, studies have shown that giving animals choices and increased control in their environment—such as the ability to choose whether to be inside or outside, or to control a light or a shower—can further improve their welfare [136,137,138,139]. With dolphins, this could be implemented by practices such as providing them with methods to explicitly choose between different training activities, enrichment items, types of reinforcers (e.g., food, rubs, toys), or to explicitly opt out of a particular activity [140,141,142].

Beyond choice and control, it has also been suggested that providing animals with situations and devices that challenge them cognitively could further improve their welfare [128,129,143,144]. Multiple studies have demonstrated that introducing cognitive challenges can lead to indications of improved welfare such as increases in movement, habitat utilization, and signs of positive excitement, along with decreases in stereotypies and other stress behaviors [145,146,147,148,149,150]. With dolphins, such cognitive challenges might come in a variety of forms, including cognitive research, puzzle devices, computer tasks, or “thinking games” (i.e., training of conceptual rules such as repeat, imitate, and innovate) [91,129,150,151,152,153,154].

## 6. Conclusions

In conclusion, the effects of the quality of the environment on animals’ cognitive and neural functioning are well-established. Numerous studies with numerous species have demonstrated detrimental effects when animals are housed in impoverished conditions [20,21,22]. The relevance of this concern to dolphins is supported by studies that have shown significant inactivity, aggression, and a head-pressing stereotypy when dolphins were kept in abnormally small, barren enclosures [44,53,89]. Thus, we agree with Jacobs et al. [18] on the importance of this concern. However, it is puzzling why, instead of pointing to these cases as individual welfare problems that clearly needed to be addressed, Jacobs et al. simply stipulate that zoos are inherently impoverished for dolphins, rather than thoughtfully engaging in the scientific question of whether this is true.

Indeed, when one does examine the data, one finds that the typical environment of dolphins in modern zoological facilities is in fact more accurately described as belonging to the standard-to-enriched side of the continuum, and that there is no evidence of wide-spread (or even common) behavioral characteristics that would suggest the effects of an inherently impoverished environment.

In addition to avoiding impoverished environments, multiple studies have documented the effects of enriching animals’ environments in various ways [19,20,21,22,23,24,25,26,27,28]. The most recent addition to this literature is the suggestion that beyond complexity, environmental elements that provide cognitive challenges can further enhance animal welfare [128,129,143]. While there have been a few studies so far exploring the welfare effects of cognitive challenge with dolphins [153,155,156], further research is warranted to determine the characteristics that make effective cognitive challenges, and to explore positive welfare benefits that might encourage more widespread adoption of such experiences into standard zoological practice.

## Figures and Tables

**Table 1:** Factors characterizing different environments on the impoverished/enriched continuum.

Environment	Factor		
	Social Situation	Enclosure Size	Environmental Complexity/ Variability
Impoverished	Alone	Small	Barren
Standard/control	With others	Small or medium	Barren
Enriched/complex	With others	Large	Complex/variable

## Data Availability

Not applicable.
